# Systemic inflammation, depression and obstructive pulmonary function: a population-based study

**DOI:** 10.1186/1465-9921-14-53

**Published:** 2013-05-15

**Authors:** Yanxia Lu, Lei Feng, Liang Feng, Ma Shwe Nyunt, Keng Bee Yap, Tze Pin Ng

**Affiliations:** 1 Gerontological Research Programme, Yong Loo Lin School of Medicine, National University of Singapore, NUHS Tower Block, 9th Floor, 1E Kent Ridge Road, 119228 Singapore, Singapore; 2 Department of Psychological Medicine, National University Hospital System, NUHS Tower Block, 9th Floor, 1E Kent Ridge Road, 119228 Singapore, Singapore; 3 Department of Geriatric Medicine, Alexandra Hospital, Singapore, Singapore

**Keywords:** Depressive symptoms, Pulmonary function, Healthy individuals, Common neurobiological process, Inverse association

## Abstract

**Background:**

Levels of Interleukin-6 (IL-6) and C-creative protein (CRP) indicating systemic inflammation are known to be elevated in chronic diseases including chronic obstructive pulmonary disease (COPD) and depression. Comorbid depression is common in patients with COPD, but no studies have investigated whether proinflammatory cytokines mediate the association between pulmonary function and depressive symptoms in healthy individuals with no known history of obstructive pulmonary diseases.

**Methods:**

In a population-based sample (n = 2077) of individuals aged 55 and above with no known history of obstructive pulmonary disease in the Singapore Longitudinal Ageing Study (SLAS), we analyzed the relationships between IL-6 and CRP, depressive symptoms (GDS-15 ≥5) and obstructive pulmonary function (FEV1% predicted and FEV1/FVC% predicted).

**Results:**

High serum levels of IL-6 and CRP were associated with greater prevalence of depressive symptoms (p < 0.05). High IL-6, high CRP and depressive symptoms were independently associated with decreased FEV1% predicted and FEV1/FVC% predicted after adjusting for smoking status, BMI and number of chronic inflammatory diseases. Increasing grades of combination of inflammatory markers and/or depressive symptoms was associated with progressive increases in pulmonary obstruction. In hierarchical models, the significant association of depressive symptoms with pulmonary obstruction was reduced by the presence of IL-6 and CRP.

**Conclusions:**

This study found for the first time an association of depressive symptoms and pulmonary function in older adults which appeared to be partly mediated by proinflammatory cytokines. Further studies should be conducted to investigate proinflammatory immune markers and depressive symptoms as potential phenotypic indicators for chronic obstructive airway disorders in older adults.

## Background

Although chronic obstructive pulmonary disease (COPD) is known to be characterized by an intense local inflammatory response in the lungs, recent research strongly implicates the role of systemic inflammation as well [1-3]. Raised levels of the representative pro-inflammatory cytokines, interleukin (IL)-6 and C-reactive protein (CRP) have been shown to be associated with low forced expiratory volume in one second (FEV1) and FEV1/FVC (forced vital capacity) indices of chronic pulmonary obstruction in two population-based studies [4,5], as well as in patients with stable-state obstructive airway disease [2,6] and during exacerbation [7]. The presence of systemic inflammation may explain the known observation that COPD is frequently found co-morbid with other systemic diseases such as atherosclerotic disease, diabetes, heart failure, chronic kidney disease, osteoporosis, sarcopenia, cognitive impairment and depression which are also characterized by raised levels of proinflammatory cytokines [1-3].

Depression is a prominent co-morbidity in COPD [8]. Levels of IL-6 and CRP have been shown to be elevated in individuals with depression [9,10] and decreased after antidepressant treatment [11]. A shared inflammatory pathway involving central and systemic responses underlying the comorbidity of obstructive pulmonary function and depression is thus possible, but has not been directly investigated in studies.

In this population-based study of community-living older adults, we investigated whether proinflammatory cytokines mediated the association between obstructive pulmonary function and depressive symptoms in healthy older adults without a known history of obstructive pulmonary diseases. We tested the hypotheses that (1) high serum levels of IL-6 and CRP were associated with a greater prevalence of depressive symptoms; (2) high serum IL-6 and CRP levels were associated with FEV1 and FEV1/FVC measures of obstructive pulmonary function; (3) depressive symptoms was inversely associated with FEV1 and FEV1/FVC measures of obstructive pulmonary function; and (4) the association of depressive symptoms with FEV1 and FEV1/FVC measures of obstructive pulmonary function was mediated by IL-6 and CRP.

## Methods

### Study design and participants

The present study involved selected participants (*n* = 2077) in the Singapore Longitudinal Ageing Study (SLAS), a prospective population-based cohort study of ageing and health. Details of the study design, sampling procedures and data collection have been previously described [12]. Briefly, a total population sample (N = 4190) of all older adults aged 55 years and over, who were residents in contiguous study localities in South East region (SLAS-1) and South Central/South West region (SLAS-2) of Singapore were identified from door-to-door census, and invited to participate in the study. Residents who were mentally or physically unable to give informed consent or participate were excluded. The response rate was 78.5%. The analytical sample in this study was drawn using baseline data from the combined SLAS-1 and SLAS-2 cohort members for a total of 2077 study participants who did not have a known history of asthma or COPD, who had technically acceptable spirometric measurements according to American Thoracic Society standards, and for whom measurements for proinflammatory cytokines were available from laboratory analyses on serum specimens. The study was approved by the National University of Singapore Institutional Review Board. All participants signed written informed consent for study participation before completing an extensive range of baseline interviews, physical examinations and testing.

### Depressive symptoms

The presence of depressive symptoms was determined by the Singapore translation of the 15-item Geriatric Depression Scale (GDS-15) [13]. The GDS was well suited for the study because it is largely free of the measurement artefact due to overlapping somatic symptoms of physical illness(es) and depression. Scores range from 0 to 15, and scores of 5 or more are indicative of depressive symptoms. In validation studies [13] in the local older population, translated versions of the GDS-15 have shown high internal (Cronbach’s alpha 0.80), test-retest (intraclass coefficient 0.83) and inter-rater reliability (coefficient 0.94).

### Pulmonary function

Pulmonary function testing was performed using a portable, battery operated, ultrasound transit-time based spirometer (Easy-One; Model 2001 Diagnostic Spirometer, NDD Medical Technologies, Zurich, Switzerland). Calibration was checked daily with a 3-L syringe. At least three acceptable forced expiratory maneuvers were performed with the respondent seated with recommended guidelines and standardization of procedures [14]. The measures of airflow obstruction were (1) forced expiratory volume, 1 sec (FEV1), expressed as a percentage value of predicted FEV1 based on age, sex, height of the individual (FEV1% predicted); (2) FEV1/FVC% expressed as a percentage of the ratio of FEV1 to forced vital capacity (FVC) based on age, sex, height of the individual (FEV1/FVC% predicted).

### Proinflammatory immune markers

Venous blood samples were collected and centrifuged for 10 min at 3,000 r/min at 4°C. The serum was subsequently removed and stored at -80°C until analysis. Serum concentrations of human CRP were determined by a specific ELISA kit (Chemicon International, Temecula, CA) according to the manufacturer's instructions. The levels of serum IL-6 were measured using commercial ELISA kit (R&D Systems, USA).

### Co-variables

Baseline data on co-variables considered to be potential cofounders included age, height, smoking status, body mass index, and number of chronic inflammatory diseases (cardiovascular disease, diabetes, rheumatoid arthritis, chronic kidney disease, osteoporosis and others).

### Statistical analyses

Data analysis was performed using the software package PASW Statistics version 18 (formerly SPSS, and currently renamed IBM SPSS). Odds ratio (OR) of association of high and low levels of IL-6 and CRP with presence of depressive symptoms were estimated with 95% confidence intervals (CIs) in chi-squared tests. Levels of IL-6 and CRP were dichotomized into high and low levels by the closest approximate median value of each variable.

The association of depressive symptoms, and IL-6 and CRP levels with pulmonary function was examined in univariate and multivariable regression analyses. To control for potential confounding influences, the latter included as covariates smoking status, BMI and number of chronic inflammatory diseases which are known to be associated with chronic systemic inflammation and determined a priori to be important influencing factors for pulmonary obstruction. To evaluate the role of IL-6 and CRP in explaining the association of depressive symptoms and pulmonary function, we included IL-6 and CRP in hierarchical models to observe their impact on depressive symptom– pulmonary function association.

We used ANCOVA (adjusting for smoking status, BMI and number of chronic inflammatory diseases) to compare differences in mean FEV1% predicted and FEV1/FVC% predicted among combination groups with low, moderate or high levels of inflammation and combination groups of high inflammation and depressive symptoms: low GDS-low inflammation, low GDS-high inflammation, high GDS-low inflammation, high GDS-high inflammation. Combined low IL-6 and low CRP were considered as low inflammation; combined high IL-6 or high CRP were considered as moderate inflammation; combined high IL-6 and high CRP were considered as high inflammation.

## Results

### Sample characteristics

The average age of the study sample was 66.3 years. Among the respondents, 36.7% were males, and 63.3% were females. The proportion of past and current smokers among respondents was 21.1%. Table 1 shows the socio-demographic characteristics, mean scores of pulmonary function, depressive symptoms, and levels of proinflammatory immune markers in study participants.

**Table 1:** Socio-demographic status, depressive symptoms, inflammatory cytokines and pulmonary function of study participants aged 55 years and over

	**N, mean**	**%, ±SD**
Total	2077	
Sex		
Male	763	36.7
Female	1314	63.3
Age (year, M ± SD)	66.30	± 7.78
Smoking		
Never smoker	1638	78.9
Past smoker	247	11.9
Present smoker	192	9.2
BMI, mean (SD)	23.61	3.68
Chronic inflammatory diseases	1400	67.4
Cardiovascular disease	1279	61.6
Diabetes	318	15.3
Rheumatoid arthritis	254	12.2
Chronic kidney disease	11	0.5
Osteoporosis, and others	67	3.2
No. of chronic inflammatory diseases	1.31	1.22
GDS Score(M ± SD)	1.07	± 2.02
GDS <5	1964	94.6
GDS ≥ 5	113	5.4
C-reactive protein (g/dL)		
Mean ± SD	22.95	± 55.1
Median, inter-quartile values	9.65	4.26, 19.64
Low CRP (≤9.65)	1206	58.1
High CRP (>9.65)	871	41.9
IL-6 (pg/ml)		
Mean ± SD	3.19	± 6.73
Median, inter-quartile values	2.00	2.00, 2.50
Low IL-6 (≤2.00)	1338	64.4
High IL-6 (>2.00)	739	35.6
FEV1 (litre, years, M ± SD)	1.81	± 0.56
FEV1 % Predicted (M ± SD)	99.22	± 23.4
FEV1/FVC% predicted (M ± SD)	86.43	± 13.3
FEV1% predicted <80%, n (%)	374	18.0
FEV1/FVC% predicted <70.0%	296	14.3

### Depressive symptoms and proinflammatory markers

A greater prevalence of GDS ≥ 5 depressive symptoms was found among participants with high levels of serum IL-6 compared with those with low levels of serum IL-6 (7.1% vs. 4.5%, χ^2^ = 6.092, OR = 1.61, 95% CI 1.10-2.37, *p* = 0.014) and among participants with high levels of CRP compared to those with low levels of CRP (6.2% vs. 4.3%, χ^2^ = 3.976, OR = 1.49, 95% CI 1.01-2.20, *p* = 0.046). See Table 2.

**Table 2:** Prevalence of depressive symptoms (GDS ≥ 5) by levels of IL-6 and CRP

	**GDS ≥ 5, n (%)**	**χ**^**2**^	**OR (95% C.I)**	***P***
Low IL-6	60 (4.5)			
High IL-6	52 (7.1)	6.092	1.61 (1.10-2.37)	0.014
Low CRP	52 (4.3)			
High CRP	54 (6.2)	3.976	1.49 (1.01-2.20)	0.046

### Depressive symptoms and pulmonary function

FEV1% predicted and FEV1/FVC% predicted were significantly lower for respondents with depressive symptoms, in comparison to respondents without depressive symptoms, and remained significant in the regression model that controlled for smoking status, BMI and number of chronic inflammatory diseases (Model 1) (Table 3).

**Table 3:** Hierarchical regression analyses of association of depressive symptoms with pulmonary function, and mediating effect of CRP and IL-6

**Independent**	**FEV1 % Predicted**					**FEV1/FVC % Predicted**				
**Variables**	**B**	**SE**	***β***	***t***	***P***	**B**	**SE**	***β***	***t***	***P***
Base model	Unadjusted									
GDS (versus <5)	-4.934	2.331	-0.047	-2.117	0.034	-4.268	2.007	-0.048	-2.127	0.034
CRP (versus low)	-4.397	1.096	-0.096	-4.010	<0.001	-4.677	1.025	-0.102	-4.564	<0.001
IL-6 (versus low)	-5.137	1.086	-0.105	-4.730	<0.001	-4.049	1.131	-0.079	-3.580	<0.001
Model 1	Adjusted for smoking, BMI, no. of chronic inflammatory diseases									
GDS (versus <5)	-4.715	2.340	-0.045	-2.015	0.044	-4.234	2.022	-0.048	-2.093	0.036
CRP (versus low)	-4.239	1.131	-0.092	-3.747	<0.001	-5.013	1.059	-0.109	-4.732	<0.001
IL-6 (versus low)	-4.901	1.096	-0.100	-4.471	<0.001	-3.949	1.147	-0.077	-3.443	0.001
Model 2	Adjusted for smoking, BMI, No. of chronic inflammatory diseases, GDS, CRP, and IL-6									
GDS (versus <5)	-3.895	2.332	-0.040	-1.670	0.095	-3.760	1.940	-0.044	-1.938	0.053
CRP (versus low)	-3.506	1.151	-0.076	-3.047	0.002	-3.017	0.874	-0.082	-3.451	0.001
IL-6 (versus low)	-3.584	1.164	-0.075	-3.079	0.002	-2.708	0.890	-0.071	-3.043	0.002

### Proinflammatory markers and pulmonary function

As shown in Table 3, FEV1% predicted and FEV1/FVC% predicted were significantly lower for respondents with high levels of serum IL-6 and CRP, in comparison to respondents with low levels of serum IL-6 and CRP, and remained significant after adjusting for smoking status, BMI and number of chronic inflammatory diseases in Model 1.

### IL-6 and CRP combinations and obstructive pulmonary function

**Figure 1:**
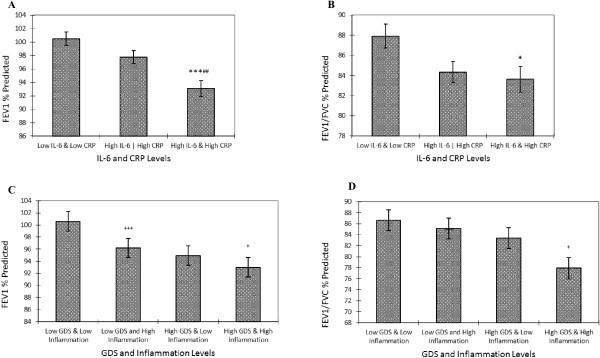
**Adjusted values of FEV1% predicted and FEV1/FVC% predicted by combined IL-6, CRP and depressive symptoms. (A) **Adjusted values of FEV1% predicted by combined IL-6 and CRP levels; **(B) **Adjusted values of FEV1/FVC% predicted by combined IL-6 and CRP levels; **(C) **Adjusted values of FEV1% predicted by combined GDS and inflammation levels; **(D) **Adjusted values of FEV1/FVC% predicted by combined GDS and inflammation levels. Adjusted: smoking, BMI, No. of chronic inflammatory diseases. High GDS (> = 5), Low GDS (less than 5); High Inflammation: high IL-6 and high CRP; Moderate Inflammation: high IL-6 or high CRP; Low Inflammation: low IL-6 and low CRP. Error bar stands for standard error. Bonferroni was used for post-hoc test. FEV1 = forced expiratory volume in 1 second; FVC = forced vital capacity; IL-6 = interleukin 6; CPR = C-reactive protein; GDS = Geriatric Depression Scale. ^*** ^p < 0.001, ^* ^p < 0.05 vs. the Low IL-6 & Low CRP group; ^##^ p < 0.01 vs. the High IL-6 | High CRP group; ^+++^ p < 0.001, ^+ ^p < 0.05 vs. the Low GDS & Low inflammation group.

As shown in Figure 1, the group with combined high IL-6-and high CRP was found to have significantly lower values of FEV1% predicted (Figure 1A) and FEV1/FVC% predicted (Figure 1B) than the groups with low IL-6-low CRP (*p* < 0.001) and high IL-6 or high CRP (*p* < 0.01).

### Combination of inflammation and depressive symptoms and obstructive pulmonary function

Participants with low GDS-high inflammation had significantly lower mean FEV1% predicted than those with low GDS-low inflammation (*p* < 0.01) (Figure 1C). Significantly lower values of FEV1/FVC% predicted were found in groups of low GDS-high inflammation and high GDS-high inflammation compared with low GDS-low inflammation group (*p* < 0.05).

### Mediational analyses

To assess whether proinflammatory immune markers mediated the association between depressive symptoms and obstructive pulmonary function, statistical mediation was tested as recommended by Stone [15]. As shown in Table 3, the introduction of IL-6 and CRP in Model 2 substantially reduced the level and significance of the association between depressive symptoms and FEV1% predicted and FEV1/FVC% predicted, while the association of IL-6 and CRP with obstructive pulmonary function remained significant.

## Discussion

This population-based study showed firstly an association between depressive symptoms and obstructive pulmonary function in apparently healthy older adults with no known obstructive pulmonary diseases. This is consistent with results from prior research based on studies of patients with asthma and COPD which showed markedly increased prevalence of depression [8,16-18]. Our results are in agreement with the findings in a population-based study of US adults [19] which observed that participants with obstructive pulmonary dysfunction were significantly more likely to present with depressive symptoms compared with controls without pulmonary dysfunction.

Secondly, we found a positive association between serum levels of IL-6 and CRP with depressive symptoms. This adds to the accumulating evidence from meta-analyses showing elevated levels of IL-6 and CRP in individuals with high levels of depressive symptoms [10] and evidence of the effects of antidepressant treatment in reducing the levels of inflammatory markers in depressive episodes [20,21].

Thirdly, serum levels of IL-6 and CRP were also found to be associated with FEV1 and FEV1/FVC measures of pulmonary obstruction. This is in agreement with the results obtained in two recent population-based studies, [4,5] as well as clinical observations of increased IL-6 and CRP levels in clinically stable patients with airway obstruction compared to healthy smoker controls [22,23]. Furthermore, IL-6 and CRP haplotypes were shown to be associated with measures of systemic inflammation and higher risk of chronic pulmonary obstruction [24]. Our study extends these findings by indicating that a combination of high IL-6 and high CRP as well as a combination of high inflammation and depressive symptoms were associated with mounting levels of pulmonary obstruction.

Our mediational analyses indicated a strong association of depressive symptoms with spirometric measures of pulmonary obstruction independent of conventional risk factors, but the significance of this association was reduced by the addition of IL-6 and CRP into the hierarchical model. These results are consonant with the hypothetical role of chronic inflammation as a shared underlying pathophysiological process in multiple organ-system diseases and functional disorders. Proinflammatory immune alterations involving central and systemic responses may be the common underlying process explaining why patients with asthma and COPD commonly present with multiple medical co-morbidities including depression, cardiovascular disorders, diabetes, obesity, chronic kidney disease, arthritis, osteoporosis and others.

IL-6 and CRP are primary proinflammatory cytokines which play important regulatory roles in the acute phase of inflammation. Studies suggest a direct role of IL-6 in airway obstruction: (1) IL-6 increases the number of CD4 cells, CD8 cells, B cells, neutrophils, and macrophages in the lung [25-27], consistent with the changes observed in human COPD pathology [28]; (2) IL-6 overexpression leads to emphysema-like airspace enlargement, peribronchiolar collections of mononuclear cells, thickening of airway walls, subepithelial fibrosis, and airway hyperresponsiveness [29]; (3) intravenous IL-6 injections into rats lead to respiratory and peripheral skeletal muscle wasting [30]; and (4) lung injury is attenuated by the absence of IL-6 after exposing animals to ozone [31]. In depression, (1) the inflammatory processes including IL-6 stimulate the release of corticotrophin-releasing hormone (CRH) and heighten hypothalamic-pituitary-adrenal axis activity, which leads to elevated cortisol levels to initiate and/or worsen symptoms of depression [32,33]; (2) central and peripheral administration of IL-6 influences mood states [34]; (3) levels of IL-6 decreases in responders to antidepressant treatment, while it remains high in nonresponders [35,36]. A distinctive clinical value of CRP is its sensitivity and ability to reveal early inflammation when other clinical parameters are equivocal [37]. Hence, in patients with chronic obstructive pulmonary disease, elevated CRP levels have been found to be associated with reduced FEV1, exercise capacity, metabolic and functional impairment, hospitalization, all-cause and COPD mortality [38-40].

Inflammatory process from airway or depression-related inflammation may hence “spill” over into the systemic circulation, promoting a generalized inflammatory reaction which may cause harm centrally and peripherally from prolonged exposure [41,42]. Our study indicates that higher levels of IL-6 and CRP combine to produce greater pulmonary obstruction and depressive symptoms. A higher index of chronic inflammation and depressive symptoms in combination were also shown to be associated with greater pulmonary obstruction, even when it was adjusted for the presence of high BMI and diagnoses of chronic inflammatory diseases. These results therefore suggest that measures of pro-inflammatory immune markers and depressive symptoms could serve as a clinically useful index in assessing and subtyping individuals for risks of obstructive pulmonary disorders.

The present study has strengths and limitations. This population-based study is largely free of clinical selection bias from over-sampling of severe cases of diseases, and also controlled for important potential confounding by age, sex, smoking, body composition and chronic inflammatory diseases in our analysis. The GDS used to assess depressive symptoms is a well validated instrument that is largely free of somatic symptoms of depression that overlap with somatic symptoms of physical illness(es). We analyzed pulmonary obstruction as a continuous variable using spirometric indices, while excluding known cases of asthma and COPD. On the other hand, the study sample very likely included a majority of COPD cases that were undiagnosed clinically. There was a higher proportion of females (63.3%) in the sample, consistent with the gender distribution of the elderly population in ageing societies in which females have longer life expectancy [43]. Gender disparity among older Singaporean adults is relatively large and the older population is therefore disproportionately female [44,45]. The higher proportions of male and depressed individuals among the non-participants might bias the results, possibly towards to the null. Spirometric measurements and laboratory analyses were available for only about half of the sample, and this may militate against the generalization of the results. Participants with technically unacceptable spirometric measures may likely include disproportionately more individuals with severe obstructive pulmonary function, and depressive symptoms, as well as possibly higher levels of inflammatory cytokines, although these data were not available. The cross-sectional study design also limits direct causal inferences from the observed associations; future studies should use longitudinal designs to assess the association of depression with proinflammatory immune profiles over time: whether changes in proinflammatory immune factors are associated with changes in pulmonary functions, and whether changes in pulmonary function over time are reflected in changes in depression levels over time.

## Conclusions

In conclusion, we found that depressive symptoms was associated with obstructive pulmonary function, and were also associated with high levels of serum IL-6 and CRP. These pro-inflammatory immune markers partially mediated the association of depressive symptoms with obstructive pulmonary function, and may delineate the neurobiological links between depressive symptoms and pulmonary function. Further studies should be conducted to better understand the role of inflammatory cytokines and the use of proinflammatory immune markers in chronic inflammatory airway diseases.

## Competing interests

The study was supported by a research grant (No. 03/1/21/17/214) from the Biomedical Research Council, Agency for Science, Technology and Research (ASTAR). The authors declare that there are no declarations of interest in relation to the current study. The authors are responsible for the writing and the content of this article.

## Authors’ contributions

YL participated in the recruitment of participants, performed the statistical analysis and drafted the manuscript. LF, LF and MSN participated in the recruitment of participants and the statistical analysis of this study. KBY participated in the design of the study and helped to draft the manuscript. TPN conceived the study, and participated in its design and coordination and helped to draft, reviewed and revised the manuscript. All authors read and approved the final manuscript.
